# Omega-3 Supplementation Prevents Short-Term High-Fat Diet Effects on the *α*7 Nicotinic Cholinergic Receptor Expression and Inflammatory Response

**DOI:** 10.1155/2021/5526940

**Published:** 2021-08-06

**Authors:** I. C. A. Martins, L. S. Contieri, C. L. Amaral, S. O. Costa, A. C. P. Souza, L. M. Ignacio-Souza, M. Milanski, A. S. Torsoni, M. A. Torsoni

**Affiliations:** ^1^Laboratory of Metabolic Disorders, School of Applied Sciences, University of Campinas, Brazil; ^2^Obesity and Comorbidities Research Center, University of Campinas, Brazil

## Abstract

The study is aimed at investigating if PUFA supplementation could prevent the effects of a short-term HFD on *α*7nAChR expression and on the severity of sepsis. Swiss mice were used for the *in vivo* experiments. For the *in vitro* experiments, we used a microglia cell line (BV-2) and a hepatoma cell line (Hepa-1c1c7) derived from mice. The animals were either fed standard chow, fed a short-term HFD (60%), or given supplementation with omega-3 fatty acid (2 g/kg or 4 g/kg bw) for 17 days, followed by a short-term HFD. Endotoxemia was induced with an intraperitoneal (i.p.) lipopolysaccharide injection (LPS, 5 or 12 mg/kg), and sepsis was induced by subjecting the animals to cecal ligation and puncture (CLP). BV-2 and Hepa-1c1c7 cells were treated with LPS (100 and 500 ng/mL, respectively) for 3 hours. RT-PCR or Western blotting was used to evaluate *α*7nAChR expression, inflammatory markers, DNMT1, and overall ubiquitination. LPS and HFD reduced the expression of *α*7nAChR and increased the expression of inflammatory markers. Omega-3 partially prevented the damage caused by the HFD to the expression of *α*7nAChR in the bone marrow and hypothalamus, decreased the inflammatory markers, and reduced susceptibility to sepsis-induced death. Exposing the BV-2 cells to LPS increased the protein content of DNMT1 and the overall ubiquitination and reduced the expression of *α*7nAChR. The inflammation induced by LPS in the BV-2 cell decreased *α*7nAChR expression and concomitantly increased DNMT1 expression and the ubiquitinated protein levels, indicating the participation of pre- and posttranscriptional mechanisms.

## 1. Introduction

Sepsis is one main causes of death in the world [1]. It derives from an intense inflammatory response of the immune system that causes damage to different organs [2]. During the development of sepsis, the systemic inflammation and the damage observed in the body are attributed to the lipopolysaccharides (LPS) in the cell walls of Gram-negative bacteria [3, 4]. LPS acts mainly through Toll-Like Receptor-4 (TLR4), which induces the activation of transcription factors and the expression of inflammatory cytokines, including tumor necrosis factor-*α* (TNF*α*), interleukin-6 (IL-6), and interferon-*γ* (IFN*γ*) [4–6].

The role of diet in the sensitivity to LPS and severity of sepsis has been investigated in the past [7, 8]. Consumption of saturated fat and cholesterol (HCD) increases the plasma concentrations of serum amyloid A and CD14, and of hepatic TLR4 mRNA [9]. Recently, Napier et al. showed that the increase in the severity and mortality of sepsis in mice fed Western diets is independent of changes to the microbiome. According to this study, the diet reprograms the baseline state of the immune system and the acute response to LPS-induced sepsis [8].

The induction of TNF*α*, IL-6, and IFN*γ* expression plays an important role in the innate immune response [5]. The delicate control of the inflammatory response to the infectious process is important to prevent damage caused by the intense production of cytokines. Alpha7 nicotinic cholinergic receptors (*α*7nAChR) participate by inhibiting the expression of inflammatory cytokines through a mechanism known as cholinergic anti-inflammatory reflex [10–12]. The binding of acetylcholine to the *α*7nAChR receptor activates the JAK2/STAT3 pathway, reducing cytokine expression [13].

It is known that overnutrition and obesity have low-grade inflammation in common [14]. The hypothalamic activation of the inflammatory nuclear factor-*κ*B (NF-*κ*B) pathway and microglial activation have been associated with obesity [15, 16]. Furthermore, hypothalamic microglia are quickly activated in response to a high-fat diet (HFD) [15] and are responsible for orchestrating the immune response and inflammation [17, 18]. Recently, we showed that a short-term HFD reduces the expression of *α*7nAChR in the hypothalamus, liver, and spleen, with significant increase in the sensitivity to and severity of LPS-induced sepsis. Moreover, hypothalamic inflammation induced by LPS is more pronounced in mice fed a HFD, but it is prevented by the intracerebroventricular administration of *α*7nAChR agonists [19].

Obese individuals show significative reduction in the expression of *α*7nAChR in adipose tissue, which, however, can be recovered with lifestyle changes and bodyweight reduction [20]. Thus, *α*7nAChR expression could be damaged by either dietary or inflammatory factors that could modulate the presence of the receptor in the membrane through different pathways. The methylation of CpG sites in the promoter region, the activation of histone deacetylase, and ubiquitination are possible mechanisms that could reduce the presence of the cholinergic receptor in the membrane [21–24].

Dietary polyunsaturated fatty acids (PUFAs) play an important role in the prevention of inflammatory damage [25, 26]. PUFA modulates the expression of cytokines and prevents the development of insulin resistance in response to a high-fat diet [27, 28]. Given the increase in the susceptibility of obese specimens to sepsis, the effect of a high-fat diet on the expression of *α*7nAChR, and the anti-inflammatory effect of PUFA, we hypothesized that PUFA supplementation could prevent the effects of a HFD on *α*7nAChR expression and on the severity of sepsis.

## 2. Materials and Methods

### 2.1. Animals

The experimental procedures involving mice were performed in accordance with the guidelines of the Brazilian Society of Laboratory Animal Science (SBCAL) and were approved by the Ethics Committee on Animal Use (ECAU) (ID protocol 41841 and 49581) of the University of Campinas (UNICAMP). All efforts were made to minimize the number of animals used. Male Swiss mice (*Mus musculus*) (8 weeks old, 30–40 g bodyweight) were provided by the Animal Breeding Center at the University of Campinas. The mice were distributed in groups of 3 or 5 individuals in a room with controlled temperature (22–24°C) and 12 h light/dark cycle, with access to water and food *ad libitum*.

### 2.2. Experimental Design

For this study, the mice were randomly divided into three groups: one group was fed a standard chow (SC) (Nuvilab® CR-1, Nuvital, PR, Brazil), another group was fed a high-fat diet (HFD; 60%) for 3 days, and the remaining group was previously supplemented orally with omega-3 fatty acid (2 g/kg or 4 g/kg body weight) for 17 days and fed a high-fat diet from the 15^th^ to the 17^th^ day (HFD*ω*3). As a control for supplementation, the SC and HFD groups were offered the same doses of water. The HFD was prepared in our laboratory according to the AIN-93G standard modified for high-fat (60%) content (Table 1). The source of omega-3 was fish oil (EPA/DHA: 5.4), donated by Naturalis®.

### 2.3. Inflammatory Response

To evaluate the inflammatory response and induce endotoxemia, the mice were injected with lipopolysaccharides (LPS—Escherichia coli serotype O111:B4. L2630, Sigma-Aldrich, St. Louis MO, USA) or saline solution (0.9%).

LPS was diluted in saline solution, and the concentration was adjusted according to the protocol (5 or 12 mg/kg). It was administered intraperitoneally, and after 2 h, the mice were sacrificed to collect the tissues (hypothalamus, spleen, liver, and bone marrow cells).

### 2.4. Cecal Ligation and Puncture (CLP) and Survival Rate

To determine the survival rate, we performed CLP to induce sepsis in all groups (SC, HFD, and HFD*ω*3). The mice in the SC, HFD, and HFD*ω*3 groups were separated into four subgroups: SC-sham (mice subjected only to laparotomy) and SC-CLP, HFD-CLP, and HFD*ω*3-CLP (mice subjected to CLP). Firstly, the mice were anesthetized with 5% isoflurane, and a 1 cm midline incision was made on the ventral surface of the abdomen to expose the cecum, which was then partially ligated at its base below the ileocecal valve with a 3-0 silk suture. Next, the cecum was punctured once with a needle (18G-1, 2 mm) on the same side of the cecum, and the fecal content was leaked into the peritoneal cavity. The sham-operated mice were submitted to an identical procedure, except for the actual cecal ligation and puncture [29]. Subsequently, the cecum was reinserted into the original position, and the abdomen was sutured. Following the procedure, the animals were fed a standard control diet, and the survival rate was recorded every 2 h until day 4.

### 2.5. Sepsis Development Markers

To characterize CLP and sepsis development, serum samples from animals of all groups (SC-SHAM, SC-CLP, HFD-CLP, and HFD*ω*3-CLP) were used for semiquantitative determination of C-Reactive Protein (CRP). The Biolatex PCR kit (Bioclin K044-1) was used. The method is based on an agglutination reaction of latex particles covered with Gamma-Globulin anti-CRP, specially treated to prevent nonspecific agglutination. Agglutination is visible in a sample with concentrations of CRP equal to or greater than 6 mg/L, according to the references established by the WHO International Standards.

In addition, animals undergoing CLP surgery showed clinical signs such as lethargy and difficulty in walking [30].

### 2.6. Tissue Extraction

All mice were anesthetized (100 mg of ketamine/kg of body weight and 100 mg of xylazine/kg of body weight, i.p.) and subsequently euthanized for the extraction of the hypothalamus, liver, and spleen; isolation of bone marrow cells; and collection of intraperitoneal macrophages. The extracted tissues were snap-frozen on dry ice for storage at −80°C until processing for qRT-PCR or Western blotting. Blood was collected after decapitation, and the samples were centrifuged at 2500 rpm for 20 min and stored at −80°C.

### 2.7. Collection of Isolated Bone Marrow Cells

The long bones (femur and tibia) were removed and placed in 0.5 mL perforated tubes overlapped with 1.5 mL tubes, which were then centrifuged at 1200 rpm for 15 sec. The cell pellet was resuspended in RPMI 1640 culture medium (Roswell Park Memorial Institute; Invitrogen Carlsbad, CA, USA) supplemented with 10% fetal bovine serum (Invitrogen) and 1% penicillin (100 U/mL)/streptomycin (100 *μ*g/mL) (Invitrogen). The cells were placed on 60 mm culture dishes and cultivated for 7 days at 37°C, in an atmosphere containing 5% CO_2_ and 95% humidity. After this period, they were collected for Western blotting, qRT-PCR, and immunofluorescence.

### 2.8. Collection of Intraperitoneal Macrophages

Peritoneal macrophages were collected in the anesthetized mice (100 mg of ketamine/kg of body weight and 100 mg of xylazine/kg of body weight, i.p.). A small incision along the midline of the mice's abdomen was made with sterile scissors. Then, a 10 mL syringe with cold PBS solution was injected in the peritoneal with a 20-G needle facing inward. After a small massage in the peritoneal cavity with the PBS solution, the fluid was aspirated from the peritoneum. The fluid recovery was ~8 mL per mice. After that, pooled peritoneal lavage fluids were centrifuged at 1200 rpm at 4° C for 15 min. The supernatant was discharged, and the pellet was washed to eliminate red blood cells. The precipitate was resuspended in RPMI supplemented with 10% fetal bovine serum and 1% antibiotic (0.1 U/mL penicillin and 0.1 mg/L streptomycin) and storage for qRT-PCR analyses.

### 2.9. Cell Culture Analysis

The microglia cell line (BV-2; RRID: CVCL_0182) derived from mice was cultivated in Dulbecco's Modified Eagle's Medium (DMEM; Invitrogen, USA), supplemented with 10% fetal bovine serum (Invitrogen, USA) and 1% penicillin (100 U/mL)/streptomycin (100 *μ*g/mL) (Invitrogen, USA), at 37°C, 5% CO_2_, and 95% humidity. The cells were treated with LPS (100 ng/mL) for 3 hours, and the protein content and the RNA were extracted for Western blotting and qRT-PCR.

The hepatoma cell line (Hepa-1c1c7; ATCC® CRL-2026™) derived from mice was cultivated in alpha Modified Eagle's Medium (*α*MEM; Invitrogen, USA), supplemented with 10% fetal bovine serum (Invitrogen, USA) and 1% penicillin (100 U/mL)/streptomycin (100 *μ*g/mL) (Invitrogen, USA), at 37°C, 5% CO_2_ and 95% humidity. The cells were treated with LPS (500 ng/mL) for 3 hours, and the protein content was extracted for Western blotting.

### 2.10. RT-PCR Analysis

The total RNA was extracted from the hypothalamus, liver, spleen, isolated bone marrow cells, intraperitoneal macrophages, and cell line BV-2 using Trizol® Reagent (Invitrogen Corporation, CA, USA) according to the manufacturer's recommendations and quantitated using a ND-2000 Nanodrop (Thermo Electron, WI, USA). Reverse transcription was performed with 3 *μ*g of the total RNA, using a High Capacity cDNA Reverse Transcription kit (Life Technologies Corporation, Carlsbad, CA, USA). The relative expression was determined using the TaqMan™ detection system, and the primers for the target genes were obtained from Applied Biosystems: Mm01312230_m1 for CHRNA7; Mm00446190_m1 for IL-6; Mm00434228_m1 for IL-1*β*; and Mm00443258_m1 for TNF*α*. GAPDH (4351309) or *β*-actin (4351315) was used as endogenous controls. Gene expression was quantitated by performing real-time PCR on an ABI Prism 7500 Fast platform. The data were analyzed using a Sequence Detection System 2.0.5 (Life Technologies Corporation, Carlsbad, CA, USA) and expressed as relative values determined by the comparative threshold cycle (Ct) method (2–*ΔΔ* Ct), according to the manufacturer's recommendations.

### 2.11. Immunofluorescence

Bone marrow-derived macrophages were plated in 24-well plates containing preflamed coverslips and washed with serum-free culture medium. After 24 hours incubated at 37°C, cells were washed once with PBS and fixed with 4% formaldehyde for 15 minutes. Then, the cells were again washed with PBS, permeabilized with 500 *μ*L of Triton ×100 0.5% for 10 minutes, blocked with a solution of BSA 3%+Triton 0.2% for 30 minutes, and incubated overnight with primary antibody of *α*7nAChR 1 : 50 (bs-1049R-Bioss Antibodies) and F4/80 1 : 200 (ab6640, Abcam). Cells were washed three times with PBS, incubated with secondary antibody Alexa 488 conjugate 1 : 500 (ab-21206, Abcam) for 1 hour and again washed with PBS three times. Then, they were stained with nuclear marker DAPI for 10 minutes and washed twice with PBS. Slides were mounted using Prolong (Invitrogen) and photographed by LeicaACTR4000 fluorescence microscopy (Leica Microsystems). ImageJ software (http://rsbweb.nih.gov/ij/) was used to count stained cells.

### 2.12. Western Blotting

The hypothalamic samples were frozen in liquid nitrogen and stored at -80°C until processing. The tissue, the isolated bone marrow cell, and cell lines BV-2 and Hepa-1c1c7 were homogenized in freshly prepared ice-cold buffer [1% (*v*/*v*) Triton X-100, 0.1 M Tris, pH 7.4, 0.1 M sodium pyrophosphate, 0.1 M sodium fluoride, 0.01 M EDTA, 0.01 M sodium vanadate, 0.002 M PMSF, and 0.01 M aprotinin]. The insoluble material was removed by centrifuging it at 12.000 rpm for 30 min at 4°C, and the protein concentration in the supernatant was determined using the Bradford dye-binding method. The supernatant was resuspended in Laemmli sample buffer and boiled for 5 min before SDS-PAGE separation using a miniature slab-gel apparatus (BioRad, Richmond, CA, USA). The electrotransfer of the proteins in the gel to a nitrocellulose membrane was performed for 120 min at 120 V. The nitrocellulose membranes were probed overnight at 4°C with specific antibodies, as described: *α*7nAChR (bs-1049R) from Bioss Antibodies (Woburn, Massachusetts USA); DNMT1 (D59A4) (#5119), p-IKK (#2697S), and p-NF-*κ*B (#3033S) from Cell Signaling (Danvers, MA, USA); and ubiquitin (sc-9133) from Santa Cruz Technology (Dallas, Texas, USA). The membranes were incubated for 2 h at room temperature with secondary antibodies (KPL, Gaithersburg, MD, USA), and the proteins recognized by them were detected via chemiluminescence (Super Signal West Pico Chemiluminescent Substrate, Thermo Fisher Scientific, MA, USA). The bands' intensities were quantified by subjecting the developed autoradiographs to optical densitometry using the Scion Image software (Scion Corp., MD, USA) and normalized for the loading control (GAPDH or *β*-actin).

### 2.13. Statistical Analysis

The data are expressed as the mean ± SD. Student's unpaired *t*-tests were used to compare the differences between two groups. One-way ANOVA was used to compare three or more categorical groups and, when necessary, Tukey's HSD test for comparison of means. The statistical significance for all analyses was set at *p* < 0.05. All statistical comparisons were performed using GraphPad Prism 8.01 (http://www.graphpad.com/scientificsoftware/prism/).

## 3. Results

**Figure 1:**
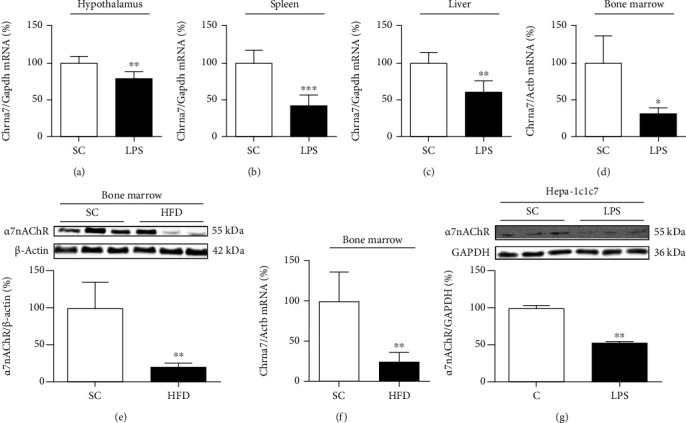
Expression of nicotinic receptors in inflammatory conditions. *Chrna7* mRNA levels in the hypothalamus (SC, *n* = 5; LPS, *n* = 5) (a); spleen (SC, *n* = 5; LPS, *n* = 5) (b); liver (SC, *n* = 5; LPS, *n* = 5) (c); and bone marrow (SC, *n* = 4; LPS, *n* = 3) (d) of mice after intraperitoneal LPS injection (12 mg/kg or 5 mg/kg of body weight). *α*7nAChR protein content (SC, *n* = 6; HFD, *n* = 4) (e) and *Chrna7* mRNA levels (SC, *n* = 4; HFD, *n* = 4) (f) in the bone marrow of mice fed a standard chow (SC) or 60% HFD for 3 days (HFD). *α*7nAChR protein levels in hepatoma cell line Hepa-1c1c7 (C, *n* = 2; LPS, *n* = 2) (g) after exposition to LPS (500 ng/mL). The expression of control (GAPDH and *β*-actin) is shown as percentages (means ± SD). Student's *t*-test analysis was used. ^∗^*p* < 0.05, ^∗∗^*p* < 0.01, and ^∗∗∗^*p* < 0.001.

Initially, the effect of inflammatory conditions on *α*7nAChR expression was evaluated. Intraperitoneally administered LPS (5 mg/kg of body weight for isolated bone marrow cells and 12 mg/kg of body weight for other tissues) significantly reduced the expression of *α*7nAChR in the hypothalamus, spleen, liver, and bone marrow (Figures 1(a)–1(d)). Furthermore, short-term HFD (3 days) also reduced the expression of *α*7nAChR in the bone marrow (Figures 1(e) and 1(f)). Additionally, in the hepatoma cell line (Hepa-1c1c7), the exposition to LPS was sufficient to reduce significantly the *α*7nAChR protein levels compared to the control cells (Figure 1(g)).

**Figure 2:**
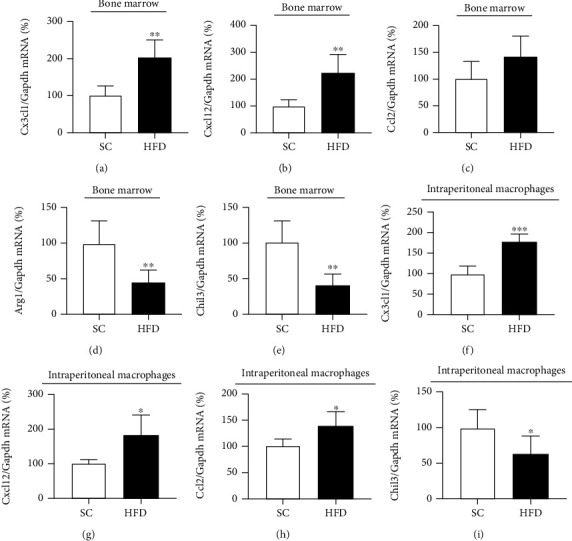
Inflammatory and anti-inflammatory markers in BMC and IP macrophages after HFD. mRNA transcript levels in the bone marrow of *CX3CL1* (SC, *n* = 5; HFD, *n* = 6) (a), CXCL12 (SC, *n* = 5; HFD, *n* = 6) (b), CCL2 (SC, *n* = 5; HFD, *n* = 6) (c), Arg1 (SC, *n* = 5; HFD, *n* = 6) (d), and Chil3 (SC, *n* = 5; HFD, *n* = 6) (e) and in intraperitoneal macrophages of *CX3CL1* (SC, *n* = 5; HFD, *n* = 5) (f), CXCL12 (SC, *n* = 5; HFD, *n* = 4) (g), CCL2 (SC, *n* = 4; HFD, *n* = 4) (h), and Chil3 (SC, *n* = 6; HFD, *n* = 6) (i) of mice fed a standard chow (SC) or 60% HFD (HFD) for 3 days. The expression of control (GAPDH) is shown as percentages (means ± SD). Student's *t*-test analysis was used. ^∗^*p* < 0.05, ^∗∗^*p* < 0.01, and ^∗∗∗^*p* < 0.001.

The expression of chemokines and markers of macrophage polarization was evaluated in bone marrow cells and intraperitoneal macrophages obtained from HFD-fed mice and control mice (Figure 2). The expression of chemokines Cx3cl1, Cxcl12, and Ccl2 was significantly higher in bone marrow cells (Figures 2(a)–2(c)) and intraperitoneal macrophages (Figures 2(f)–2(h)) of HFD-fed mice than in control mice. On the other hand, short-term HFD (3 days) significantly reduced the expression of Chil3 mRNA in bone marrow cells and peritoneal macrophages (Figures 2(e) and 2(i)) and Arg1 mRNA in bone marrow cells (Figure 2(d)).

**Figure 3:**
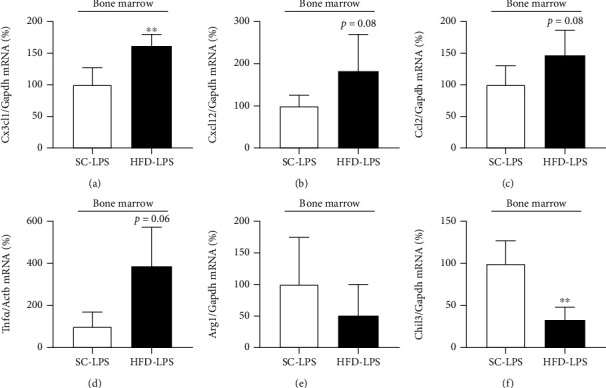
Inflammatory and anti-inflammatory markers in BMC after HFD and LPS challenge. mRNA transcript levels in bone marrow of *CX3CL1* (SC-LPS, *n* = 5; HFD-LPS, *n* = 5) (a), CXCL12 (SC-LPS, *n* = 5; HFD-LPS, *n* = 5) (b), CCL2 (SC-LPS, *n* = 5; HFD-LPS, *n* = 5) (c), TNF*α* (SC-LPS, *n* = 3; HFD-LPS, *n* = 3) (d), Arg1 (SC-LPS, *n* = 4; HFD-LPS, *n* = 5) (e), and Chil3 (SC-LPS, *n* = 5; HFD-LPS, *n* = 5) (f) of mice fed a standard chow (SC) or 60% HFD (HFD) for 3 days and injected intraperitoneally with LPS (12 mg/kg). The expression of control (GAPDH and *β*-actin) is shown as percentages (means ± SD). Student's *t*-test analysis was used ^∗∗^*p* < 0.01.

**Figure 4:**
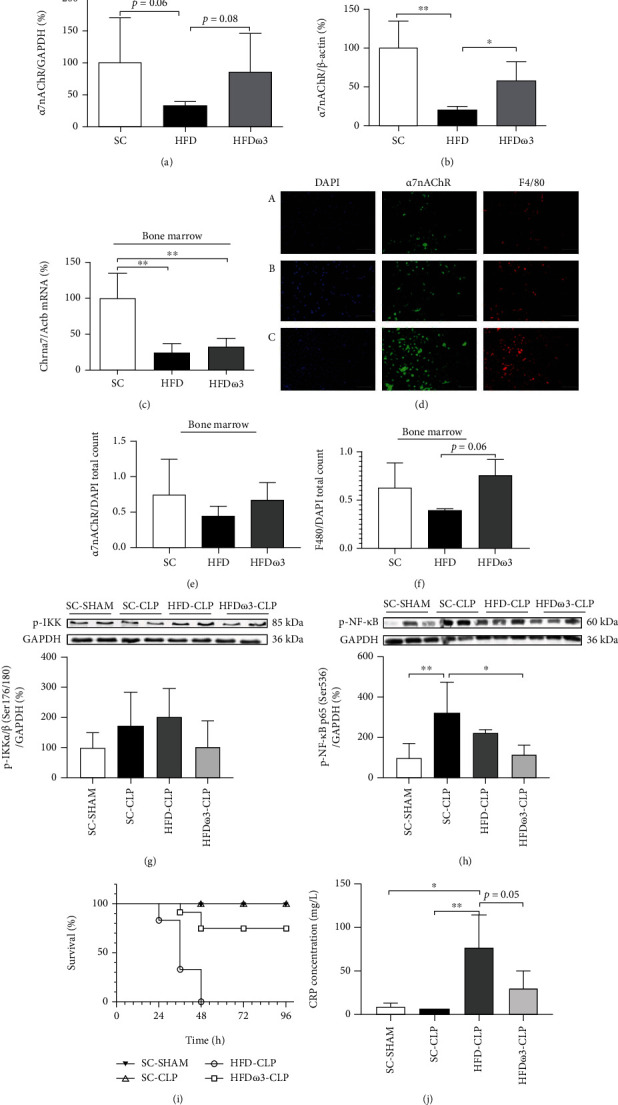
Expression of nicotinic receptors and severity of sepsis after omega-3 supplementation. *α*7nAChR protein levels in the (a) hypothalamus (SC, *n* = 6; HFD, *n* = 5; HFD*ω*3, *n* = 6) and (b) bone marrow cells (SC, *n* = 6; HFD, *n* = 4; HFD*ω*3, *n* = 6); (c) Chrna7 mRNA levels in the bone marrow cells (SC, *n* = 4; HFD, *n* = 4; HFD*ω*3, *n* = 4); (d) representative imagens *α*7nAChR+ cells (*α*-bgt-green) and F4/80+ cells (red) and nuclear labelling (DAPI-blue) (A—SC, *n* = 3; B—HFD, *n* = 3; C—HFD *ω*3, *n* = 4); (e) total count of *α*7nAChR (SC, *n* = 4; HFD, *n* = 4; HFD*ω*3, *n* = 4) and (f) total count of F4-80 (SC, *n* = 4; HFD, *n* = 3; HFD*ω*3, *n* = 4) of mice fed a standard chow (SC) or 60% HFD (HFD) for 3 days and previously supplemented with omega-3 (2 g/kg or 4 g/kg of body weight) for 17 days (HFD*ω*3). (g) p-IKK (SC-SHAM, *n* = 5; SC-CLP, *n* = 4; HFD-CLP, *n* = 5; HFD*ω*3, *n* = 5), (h) p-NF-*κ*B p65 (SC-SHAM, *n* = 5; SC-CLP, *n* = 4; HFD-CLP, *n* = 5; HFD*ω*3, *n* = 5) protein content in the hypothalamus, (i) survival rate (SC-SHAM, *n* = 5; SC-CLP, *n* = 5; HFD-CLP, *n* = 6; HFD*ω*3, *n* = 6), and (j) serum CRP concentration (SC-SHAM, *n* = 3; SC-CLP, *n* = 3; HFD-CLP, *n* = 4; HFD*ω*3, *n* = 4) of mice fed a standard chow (SC-CLP) or 60% HFD for 3 days (HFD-CLP) and previously supplemented with omega-3 for 17 days (HFD*ω*3-CLP) following CLP surgery. The expression of control (GAPDH and *β*-actin) is shown as percentages (means ± SD). In (a, b), Student's *t*-test analysis was used. In (c, e–h, and j), one-way ANOVA was used. In (i), logrank test was used. Tukey's HSD test was used for comparison of means. ^∗^*p* < 0.05, ^∗∗^*p* < 0.01, and ^∗∗∗^*p* < 0.001.

Intraperitoneal administration of LPS (12 mg/kg) to HFD-fed and control mice resulted in higher expression of Cx3cl1 and a nonsignificant increase in TNF*α* expression (*p* = 0.06). Cxcl12 and Ccl12 mRNA levels in bone marrow cells obtained from HFD-fed mice were similar to bone marrow cells obtained from control mice (Figures 3(a)–3(d)). However, Chil3 expression after intraperitoneal administration of LPS was lower in bone marrow cells of HFD-fed than in control mice (Figure 3(f)). Arg1 mRNA level was not different between HFD-fed and control mice (Figure 3(e)). Previous supplementation with *ω*3 (EPA and DHA) for 17 days partially prevented the effects of the short-term HFD. As we can see in Figure 4(a), the hypothalamic expression of *α*7nAChR was reduced by the HFD (3d) consumption (*p* = 0.06). The previous supplementation with *ω*3 seems to mitigate the effect of HFD. In bone marrow cells, although mRNA levels were not different between HFD and HFD*ω*3, the supplementation significantly prevented the effect of HFD on the *α*7nAChR protein levels (Figures 4(b)–4(c)), suggesting the modulation of posttranslational mechanisms, such as proteasomal degradation after ubiquitination.

The effect of supplementation was also evaluated through immunofluorescence of bone marrow cells. As can be observed in Figures 4(d)–4(f) the number of F4/80^+^ and *α*7nAChR^+^ cells was similar in HFD and HFD*ω*3 compared to control mice. However, the number of F4/80^+^ cells in HFD*ω*3 mice is closer to the control than observed in HFD mice (HFD vs. HFD*ω*3, *p* = 0.06).

The severity of sepsis-induced via cecal ligation and puncture (CLP) was evaluated in the HFD-fed mice and compared to the mice in the HFD*ω*3 group (Figure 4). Firstly, hypothalamic p-IKK and p-NF-*κ*B levels were evaluated after sepsis-induced via CLP. The hypothalamic p-IKK levels were not different after CLP in the SC and HFD groups compared to the sham group (Figure 4(g)). On the other hand, CLP significantly increased the hypothalamic p-NF-*κ*B levels in the SC-CLP group compared to the SC-SHAM group (3-fold). The hypothalamic p-NF-*κ*B levels in the HFD-CLP group were similar to those in the SC-CLP group. The supplementation of the mice in the HFD group with *ω*3 (HFD*ω*3-CLP) prevented NF-*κ*B activation induced by CLP, resulting in similar values to those of the mice in the SC-SHAM group (Figure 4(h)). These effects were accompanied by higher susceptibility to sepsis-induced death of HFD mice compared to the control mice (HFD-CLP vs. SC-CLP), since all HFD mice died. However, this effect was significantly reduced by previous supplementation with *ω*3 (HFD*ω*3 -CLP), considering that no mice died after 48 hours of analysis (Figure 4(i)). To confirm the development of sepsis, we quantified the concentration of CRP in the serum. The HFD-CLP and HFD*ω*3-CLP groups showed greater concentration compared to the SC-SHAM and SC-CLP groups, but the supplemented group had a smaller amount compared to the HFD group (Figure 4(j)).

**Figure 5:**
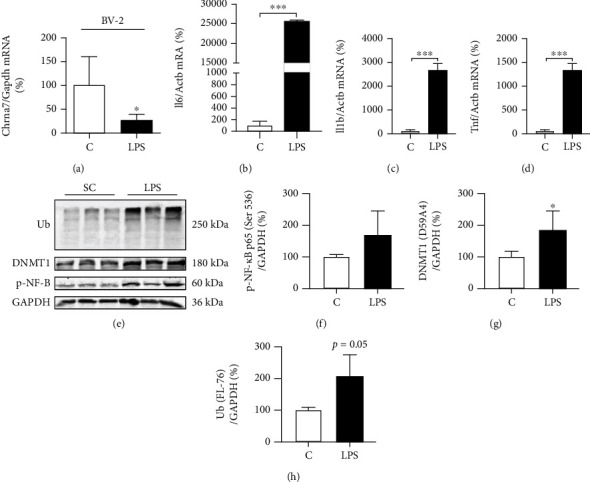
Inflammatory markers in the BV-2 cell line. mRNA transcript levels of *Chrna7* (C, *n* = 5; LPS, *n* = 6) (a), IL-6 (C, *n* = 3; LPS, *n* = 3) (b), IL-1*β* (C, *n* = 3; LPS, *n* = 3) (c), and TNF*α* (C, *n* = 3; LPS, *n* = 3) (d) cytokines in BV-2 cells treated with LPS (100 ng/mL); Western blotting (e) and relative quantification of p-NF-*κ*B p65 (Ser 536) (C, *n* = 3; LPS, *n* = 3) (f), DNMT1 (D59A4) (C, *n* = 5; LPS, *n* = 5) (g), and ubiquitin (Ub—FL-76) (C, *n* = 3; LPS, *n* = 3) (h) in BV-2 cells after LPS treatment (100 ng/mL). The expression of control (GAPDH and *β*-actin) is shown as percentages (means ± SD). Student's *t*-test analysis was used. ^∗^*p* < 0.05 and ^∗∗∗^*p* < 0.001.

In order to perform the *in vitro* simulation of the presence of cytokines after CLP, the BV-2 cells were exposed to LPS for 3 hours. As shown in Figures 5(a)–5(d), LPS (100 ng/mL) treatment significantly reduced the levels of *α*7nAChR mRNA (Figure 5(a)) and increased the amount of mRNA transcript in IL-6, IL-1*β*, and TNF*α* (Figures 5(b)–5(d)) compared to the control cells. Subsequently, we evaluated p-NF-*κ*B, DNMT1, and the overall ubiquitination in the BV-2 cells after exposition to LPS (Figures 5(e)–5(h)). LPS seems to have increased the p-NF-*κ*B levels compared to the control cells (Figures 5(e) and 5(f)); the DNMT1 levels were 70% higher in cells exposed to LPS in relation to the control cells (Figures 5(e) and 5(g)), and the total protein ubiquitination also increased (2-fold) after exposition to LPS (Figures 5(e) and 5(h)).

## 4. Discussion

The inflammatory response is an important mechanism to protect the body against pathogens. However, its intensity must be regulated to avoid damages to cellular structures and to the physiological process responsible for maintaining homeostasis. Different cell types and molecules participate in stimulating and inhibiting inflammatory pathways and components of the immunological system. *α*7nAChR is one of these components, and it acts by reducing the expression of inflammatory cytokines [10, 12]. Recently, we showed that hypothalamic *α*7nAChR expression was reduced in rodents subjected to a short-term HFD, which rendered them more responsive to sepsis. However, ICV administration of PNU, a selective agonist of *α*7nAChR, reduced the inflammatory response [19]. Thus, although *α*7nAChR plays an important role in the control of the inflammatory response, we hypothesize that both the nutritional components of a HFD and inflammatory milieu impair the expression of this receptor, while PUFA protects it.

In this study, we investigated this hypothesis and evaluated a possible mechanism associated with the reduced expression of *α*7nAChR. Initially, we showed that intraperitoneal administration of LPS reduced the expression of *α*7nAChR in the hypothalamus, spleen, liver, and bone marrow. A previous study also showed that a HFD had a similar effect on *α*7nAChR expression [19]. Additionally, LPS treatment also significantly reduced the expression of *α*7nAChR in hepatoma (Hepa-1c1c7) and microglial (BV-2) cell cultures. Both models, HFD consumption and LPS exposition, trigger inflammatory signals associated with cellular damage. Here, we showed that HFD consumption for three days was sufficient to increase the expression of inflammatory markers in bone marrow cells (Cx3cl1 and Cxcl12) and intraperitoneal macrophages (Cx3cl1, Cxcl12, and Ccl2). On the other hand, the expression of *Chil3* and *Arg1* was reduced in HFD compared to SC mice, suggesting the M1 polarization of macrophages after short-term HFD consumption.

To investigate this idea, we measured the expression of inflammatory markers in bone marrow cells after LPS exposition. *Cx3cl1* and *TNFα* expression increased significantly in bone marrow cells of HFD mice compared to SC mice after exposition to LPS. *TNFα* and *Cx3cl1* can affect the expression of each other increasing inflammation [31]. Furthermore, Friggeri and colleagues showed that the level of *Cx3cl1* expression was inversely proportional to mortality in patients with sepsis [32], suggesting that this chemokine has an important role in the severity of sepsis and mortality. Interestingly, previous studies showed that the activation of *α*7nAChR receptor has an important role in the M1/M2 polarization of BV-2 microglia [33] and lung macrophage [34]. Therefore, short-term HFD consumption or inflammatory pathway activation, as demonstrated after LPS administration, reduces *α*7nAChR expression in immune cells leading to increased inflammatory potential. The activation of hypothalamic inflammatory signaling within 1 to 3 days after the start of a HFD has been previously demonstrated [15], suggesting that damage to *α*7nAChR of expression may have early onset. In adipocytes of obese subjects, *α*7nAChR was downregulated, but a lifestyle intervention that promoted weight loss also increased *α*7nAChR expression [20]. These data suggest that the presence of proinflammatory factors associated with diet and obesity can be responsible for reducing the expression of *α*7nAChR.

The use of unsaturated fatty acids is currently being explored as a therapeutic strategy to prevent metabolic diseases due to their anti-inflammatory role [25]. The previous supplementation of mice with fish oil containing an EPA/DHA mixture partially prevented the effects of HFD consumption in the expression of *α*7nAChR and CLP-induced sepsis. EPA/DHA supplementation seems to improve the *α*7nAChR levels in both the hypothalamus and bone marrow cells, although in bone marrow cells the transcript levels of supplemented mice did not change. These results suggest that in bone marrow cells, posttranslational mechanisms such as ubiquitination and proteasomal degradation may be reducing the amount of *α*7nAChR protein in HFD mice. However, although omega-3 supplementation appears to prevent this process, it has no effect on the impairment of gene expression caused by HFD consumption. Furthermore, NF-*κ*B phosphorylation induced by HFD was significantly reduced by previous EPA/DHA supplementation. NF-*κ*B is a transcription factor associated with the expression of inflammatory cytokines and immunometabolic disorders [14], suggesting that the improvement in *α*7nAChR expression can be associated with the reduction in the inflammatory environment. EPA and DHA play key roles in regulating homeostasis as precursors to the biosynthesis of anti-inflammatory eicosanoids, which could protect the body against inflammatory diseases [35].

Although protein synthesis may be affected by many pre- and posttranscriptional mechanisms, DNA methylation and protein ubiquitination seem to play an important role in the expression of nicotinic cholinergic receptors, particularly *α*7nAChR. Previous studies have shown that the promoter region of the *α*7nAChR gene (*Chrna7*) is rich in CpG islands, which are targets of DNA methyltransferases [21, 23]. Additionally, the assembly efficiency and the number of mature nicotinic acetylcholine receptors in the cellular membrane are regulated by the ubiquitin-proteasome system [22, 36]. DNMT1 expression increased after the exposition of BV-2 cell to LPS, suggesting the participation of the methylation process in the reduction of *α*7nAChR expression. Furthermore, LPS also stimulates NF-*κ*B activation and the expression of cytokines (TNF*α*, IL-6, and IL-1*β*) in BV-2 cells. In the central nervous system, microglia are the major LPS-responsive cells, acting through Toll-Like Receptor-4 (TLR4), which activates NF-*κ*B to generate inflammatory cytokines. In addition, NF-*κ*B has also been associated with the upregulation of DNMT1 in studies that investigated the inflammatory pathway in cancer [37, 38]. Thus, the reduction in the expression of *α*7nAChR could be associated with the activation of inflammatory pathways that lead to NF-*κ*B phosphorylation and stimulate DNMT1 expression.

Another mechanism explored to explain the reduction in *α*7nAChR expression is the degradation of proteins promoted by the ubiquitin-proteasome system (UPS). This system contributes to the degradation of intracellular misfold proteins via cellular proteostasis. LPS-induced neuroinflammation has been previously associated with increased catalytic proteasomal activity [39]. Thus, although we did not evaluate the specific ubiquitination of *α*7nAChR, the BV-2 cells exposed to LPS showed higher levels of ubiquitin than the control cells, suggesting that the ubiquitination process was stimulated by LPS.

LPS and HFD impair *α*7nAChR expression, possibly via pre- and posttranscriptional mechanisms that are activated by inflammatory conditions. These processes are triggered early after the beginning HFD consumption, contributing to additional inflammatory damages to the cells, but they can be prevented by EPA/DHA supplementation.

## Figures and Tables

**Table 1:** Nutritional composition of the high-fat diet and standard chow diet.

	Standard chow diet^1^	High-fat diet (60%)
Net protein (g%)	22.5	26.0
Fat (g%)	4.5	35.0
Carbohydrates (g%)	55.0	26.0
Crude fiber (g%)	8.0	6.0
Ashes (g%)	10.0	7.0
Total	100.0	100.0
kcal/g	3.5	5.2

Protein (kcal%)^2^	25.7	19.9
Fat (kcal%)^2^	11.5	60.2
Carbohydrate (kcal%)^2^	62.8	19.9

^1^(NUVILAB® Cr-1, Nuvital, PR, Brazil). ^2^Protein and carbohydrate = 4 kcal/g; fat = 9 kcal/g.

## Data Availability

The data will be available to interested parties through the google drive after the publication of the manuscript when requested to the corresponding author.

## Abbreviations

ANOVA: Analysis of variance
Bw: Body weight
CD14: Cluster of differentiation 14
*Chrna7*: Protein coding gene for cholinergic receptor nicotinic alpha 7 subunit
CLP: Cecal ligation puncture procedure
CRP: C-reactive protein
Ct: Comparative threshold cycle
DHA: Docosahexaenoic acid
DMEM: Dulbecco's Modified Eagle's Medium
DNMT1: DNA (cytosine-5)-methyltransferase 1
EPA: Eicosapentaenoic acid
GAPDH: Glyceraldehyde 3-phosphate dehydrogenase
HCD: High-cholesterol diet
HFD: High-fat diet
HFD-CLP: High-fat diet+CLP surgery
HFD*ω*3: High-fat diet+*ω*3 supplementation
HFD*ω*3-CLP: High-fat diet+*ω*3 supplementation+CLP surgery
i.p.: Intraperitoneal
IFN*γ*: Interferon-*γ*
IL-1*β*: Interleukin-1*β*
IL-6: Interleukin-6
JAK2: Janus Kinase 2
LPS: Lipopolysaccharide
NF-*κ*B: Factor nuclear kappa B
p-IKK: I*κ*B kinase phosphorylated
PMSF: Phenylmethylsulfonyl fluoride
p-NF-*κ*B: Factor nuclear kappa B phosphorylated
PUFA: Polyunsaturated fatty acids
qRT-PCR: Reverse transcription polymerase chain reaction quantitative real time
Rpm: Rotations per minute
RPMI: Roswell Park Memorial Institute culture medium
SC: Standard chow
SC-CLP: Standard chow+CLP
SC-SHAM: Standard chow+laparotomy
SD: Standard deviation
STAT3: Signal transducer and activator of transcription 3
TLR4: Toll-like receptor 4
TNF*α*: Tumor necrosis factor alpha
UPS: Ubiquitin-proteasome system
V: Volts
*α*7nAChR: Alpha7 nicotinic cholinergic receptors
*α*MEM: Alpha Modified Eagle's Medium
*β*-Actin: Beta-actin.

## References

[1] Rudd K. E., Johnson S. C., Agesa K. M. (2020). Global, regional, and national sepsis incidence and mortality, 1990-2017: analysis for the Global Burden of Disease Study. *The Lancet*.

[2] Singer M., Deutschman C. S., Seymour C. W. (2016). The third international consensus definitions for sepsis and septic shock (Sepsis-3). *JAMA*.

[3] Zhang G., Ghosh S. (2000). Molecular mechanisms of NF-*κ*B activation induced by bacterial lipopolysaccharide through toll-like receptors. *Journal of Endotoxin Research*.

[4] Poltorak A., He X., Smirnova I. (1998). Defective LPS signaling in C3H/HeJ and C57BL/10ScCr mice: mutations in Tlr4 gene. *Science*.

[5] Tracey K. J., Lowry S. F. (1990). The role of cytokine mediators in septic shock. *Advances in Surgery*.

[6] van Amersfoort E. S., van Berkel T. J. C., Kuiper J. (2003). Receptors, mediators, and mechanisms involved in bacterial sepsis and septic shock. *Clinical Microbiology Reviews*.

[7] Moreira A. P., Texeira T. F., Ferreira A. B., do Carmo Gouveia Peluzio M., de Cássia Gonçalves Alfenas R. (2012). Influence of a high-fat diet on gut microbiota, intestinal permeability and metabolic endotoxaemia. *The British Journal of Nutrition*.

[8] Napier B. A., Andres-Terre M., Massis L. M. (2019). Western diet regulates immune status and the response to LPS-driven sepsis independent of diet-associated microbiome. *Proceedings of the National Academy of Sciences of the United States of America*.

[9] Huang H., Liu T., Rose J. L., Stevens R. L., Hoyt D. G. (2007). Sensitivity of mice to lipopolysaccharide is increased by a high saturated fat and cholesterol diet. *Journal of Inflammation*.

[10] Tracey K. J. (2002). The inflammatory reflex. *Nature*.

[11] Gallowitsch-Puerta M., Tracey K. J. (2005). Immunologic role of the cholinergic anti-inflammatory pathway and the nicotinic acetylcholine 7 receptor. *Annals of the New York Academy of Sciences*.

[12] Pavlov V. A., Tracey K. J. (2005). The cholinergic anti-inflammatory pathway. *Brain, Behavior, and Immunity*.

[13] de Jonge W. J., van der Zanden E. P., The F. O. (2005). Stimulation of the vagus nerve attenuates macrophage activation by activating the Jak2-STAT3 signaling pathway. *Nature Immunology*.

[14] Hotamisligil G. S. (2017). Inflammation, metaflammation and immunometabolic disorders. *Nature*.

[15] Thaler J. P., Yi C. X., Schur E. A. (2012). Obesity is associated with hypothalamic injury in rodents and humans. *The Journal of Clinical Investigation*.

[16] Mendes N. F., Kim Y. B., Velloso L. A., Araújo E. P. (2018). Hypothalamic microglial activation in obesity: a mini-review. *Frontiers in Neuroscience*.

[17] Valdearcos M., Douglass J. D., Robblee M. M. (2017). Microglial inflammatory signaling orchestrates the hypothalamic immune response to dietary excess and mediates obesity susceptibility. *Cell Metabolism*.

[18] Lee C. H., Suk K., Yu R., Kim M. S. (2020). Cellular contributors to hypothalamic inflammation in obesity. *Molecules and cells*.

[19] Souza A. C. P., Souza C. M., Amaral C. L. (2019). Short-term high-fat diet consumption reduces hypothalamic expression of the nicotinic acetylcholine receptor *α*7 subunit (*α*7nAChR) and affects the anti-inflammatory response in a mouse model of Sepsis. *Frontiers in Immunology*.

[20] Cancello R., Zulian A., Maestrini S. (2012). The nicotinic acetylcholine receptor *α*7 in subcutaneous mature adipocytes: downregulation in human obesity and modulation by diet-induced weight loss. *International Journal of Obesity*.

[21] Canastar A., Logel J., Graw S. (2012). Promoter methylation and tissue-specific transcription of the *α*7 nicotinic receptor gene, CHRNA7. *Journal of Molecular Neuroscience*.

[22] Rezvani K., Teng Y., De Biasi M. (2010). The ubiquitin-proteasome system regulates the stability of neuronal nicotinic acetylcholine receptors. *Journal of Molecular Neuroscience*.

[23] Dyrvig M., Mikkelsen J. D., Lichota J. (2019). DNA methylation regulates *CHRNA7* transcription and can be modulated by valproate. *Neuroscience Letters*.

[24] Nakamura Y., Kimura S., Takada N. (2020). Stimulation of toll-like receptor 4 downregulates the expression of *α*7 nicotinic acetylcholine receptors via histone deacetylase in rodent microglia. *Neurochemistry International*.

[25] Calder P. C., Ahluwalia N., Brouns F. (2011). Dietary factors and low-grade inflammation in relation to overweight and obesity. *British Journal of Nutrition*.

[26] de Boer A. A., Monk J. M., Liddle D. M. (2016). Fish-oil-derived n-3 polyunsaturated fatty acids reduce NLRP3 inflammasome activity and obesity-related inflammatory cross-talk between adipocytes and CD11b^+^ macrophages. *The Journal of Nutritional Biochemistry*.

[27] Chacińska (2019). The impact of omega-3 fatty acids supplementation on insulin resistance and content of adipocytokines and biologically active lipids in adipose tissue of high-fat diet fed rats. *Nutrients*.

[28] O’Mahoney L. L., Matu J., Price O. J. (2018). Omega-3 polyunsaturated fatty acids favourably modulate cardiometabolic biomarkers in type 2 diabetes: a meta-analysis and meta-regression of randomized controlled trials. *Cardiovascular Diabetology*.

[29] Wen H. (2013). Sepsis induced by cecal ligation and puncture. *Methods in Molecular Biology*.

[30] Toscano M. G., Ganea D., Gamero A. M. (2011). Cecal ligation puncture procedure. *Journal of Visualized Experiments*.

[31] Jones B. A., Riegsecker S., Rahman A. (2013). Role of ADAM-17, p38 MAPK, cathepsins, and the proteasome pathway in the synthesis and shedding of fractalkine/CX3CL1 in rheumatoid arthritis. *Arthritis and Rheumatism*.

[32] for the MIP Rea Study Group, Friggeri A., Cazalis M. A. (2016). Decreased CX3CR1 messenger RNA expression is an independent molecular biomarker of early and late mortality in critically ill patients. *Critical Care*.

[33] Zhang Q., Lu Y., Bian H., Guo L., Zhu H. (2017). Activation of the *α*7 nicotinic receptor promotes lipopolysaccharide-induced conversion of M1 microglia to M2. *American Journal of Translational Research*.

[34] Pinheiro N. M., Santana F. P., Almeida R. R. (2017). Acute lung injury is reduced by the *α*7nAChR agonist PNU-282987 through changes in the macrophage profile. *The FASEB Journal*.

[35] Saini R. K., Keum Y. S. (2018). Omega-3 and omega-6 polyunsaturated fatty acids: dietary sources, metabolism, and significance – a review. *Life Sciences*.

[36] Christianson J. C., Green W. N. (2004). Regulation of nicotinic receptor expression by the ubiquitin-proteasome system. *The EMBO Journal*.

[37] Wang B., Cui Z., Zhong Z. (2016). The role and regulatory mechanism of IL-1*β* on the methylation of the NF2 gene in benign meningiomas and leptomeninges. *Molecular Carcinogenesis*.

[38] Zhang B. G., Hu L., Zang M. D. (2016). Helicobacter pyloriCagA induces tumor suppressor gene hypermethylation by upregulating DNMT1 via AKT-NF*κ*B pathway in gastric cancer development. *Oncotarget*.

[39] Pintado C., Gavilán M. P., Gavilán E. (2012). Lipopolysaccharide-induced neuroinflammation leads to the accumulation of ubiquitinated proteins and increases susceptibility to neurodegeneration induced by proteasome inhibition in rat hippocampus. *Journal of Neuroinflammation*.

